# African Urban Sexualities After *Queer Visibilities*

**DOI:** 10.1007/s12132-023-09496-w

**Published:** 2023-05-01

**Authors:** Andrew Tucker

**Affiliations:** African Centre for Cities, University of Cape Town, Cape Town, South Africa

**Keywords:** Sexuality, Non-heteronormativity, Urban, Africa, Queer

## Abstract

This article outlines why there exist important opportunities to think through what research on African urban sexualities—and specifically non-heteronormative sexualities—may mean moving forward. By looking back at the text *Queer Visibilities* that largely focused on articulating some of the relationships between the urban and sexuality over a decade ago in Cape Town, this article suggests at least three key opportunities in which future scholarship may wish to explore African urban sexualities in the current moment. These opportunities circulate around new theoretical insights that emerge from the South that may speak to but are not beholden to theories from the North, the urgent need for further empirical work on the ways sexuality interfaces with urbanisation dynamics on the continent, and to think through and give space to broader approaches to document the relationship between sexuality and the urban that include but also extend beyond more ‘traditional’ social science methods. This article then explores these opportunities in relation to the interventions that follow in this special issue.

## Introduction

This article and the collection of invited interventions that follow have been organised to bring together emergent thought on the topic of African urban sexualities, and specifically non-heteronormative sexualities. It therefore also implicitly, and sometimes explicitly, asks the question: What does it mean—and why is it important—to study or examine African urban sexualities (and especially non-heteronormative sexualities) today?

This may appear to be an obvious question to some—and perhaps one that does not, on initial reflection, need asking. After all, there has been a relative explosion of research on same-sex sexualities and growing work also on trans issues in the global South and especially Africa over the past two decades (see for example, Awondo et al., 2012; Baral et al., 2009; Bourne et al., 2016; Boyd, 2013; Camminga, 2019; Cheney, 2012; Currier, 2012; De Vos, 2007; Epprecht, 2013; Gaudio, 2009; Hendricks, 2016; Judge et al., 2009; M’Baya, 2013; Matebini et al., 2018; Msibi, 2019; Reid, 2013; Rink, 2013; Semugoma et al., 2012; Tamale, 2011). There has also been a concurrent explosion of literature on the urban South—driven in large part by the southern turn in knowledge production, especially in and on Africa (see for example, Bhan, 2019; Caldeira, 2017; Kimari, 2020; Lancione and McFarlane, 2021; Nuttall and Mbembe, 2004; Parnell and Oldfield, 2014; Parnell and Pieterse, 2014; Pieterse, 2008; Robinson, 2022; Robinson and Roy, 2016; Roy, 2011; Selmeczi, 2014; Simone, 2004; Thieme, 2018). However, as this article by way of introduction to the interventions that follow wishes to argue, scholarship has yet to consider with much sustained engagement what these two bodies of literature or research foci have meant for each other or how they can be put into conversation. A special issue framed around African urban sexualities may therefore be seen as a timely intervention that attempts to combine and further the interests of two different and sometimes distinct groups of scholars. As this article and the interventions that follow all explore, there are some exciting synergies that can be forged by linking research on urban Africa to research specifically on non-heteronormative sexualities.

This article begins by setting out why a more concerted focus on African urban sexualities and specifically work non-heteronormative groups and communities in urban spaces across the continent may be germane going forward. It then, somewhat elliptically, looks back at a text I wrote, *Queer Visibilities* (2009) over a decade ago which attempted to explore how the urban may be brought into conversation with questions related to sexuality in the post-apartheid city of Cape Town. While Cape Town is clearly not representative of the wider African continent, and the world has clearly moved on since 2009, the reason for this excavation of this text is to highlight, on the one hand, the key opportunities that can arise when studying sexualities in African urban contexts and how such empirical research can contribute to southern urban knowledges, while on the other hand also allowing for a consideration as to how much still needs to be uncovered and explored about an emerging field. The last part of this article then explores how scholars may wish to go about uncovering new ways of conceptualising, theorising and researching African urban sexualities, by pointing towards the interventions that follow.

## Why a Study of African Urban Sexualities Is both Timely and Vital

There is little doubt that over the past two decades there has been a significant growth in studies that have explored sexualities—and specifically of interest here, non-heteronormative sexualities—across Africa. While the majority of studies still hail from South Africa (and more on this later), there has also been a growth of work across the wider continent. From a range of disciplines including sociology, history, literary studies, anthropology and public health, we have seen a concerted interest in the study of non-heteronormative groups and communities. This is a far cry from the situation a few decades ago, a time when some scholars in prestigious journals when discussing sexuality still appeared surprised and somewhat taken aback to discover that concepts such as same-sex desire and the practices that led on from them were indeed prevalent across the continent.^1^

This growth of studies across the continent has also, to a large extent, been focused on individuals and communities that exist within urban environments. Yet while ‘the urban’ has been present, implicitly, in many studies, often as a backdrop or set of geographic coordinates to help locate studies, less consideration I would argue has been given to considering the actual relationship between the urban, urbanisation and non-heteronormative sexualities. This is perhaps surprising for several reasons.

First, it is surprising considering the large body of work that has also emerged over the past 20 years on the needs to consider the diverse ways in which urban processes are unfolding, the speed with which such processes are unfolding, and the intended and unintended consequences of such processes across the continent. As readers of this journal are well aware, Africa is today urbanising at an unprecedented rate (Parnell and Pieterse, 2014) and the implications this has for sustainable and equitable development are also immense (Parnell and Oldfield, 2014). Between 2020 and 2050, the African continent’s population will double, and two-thirds of that growth will occur in cities (OECD/SWAC, 2020). It is projected that by 2050 cities in Africa will have added an additional 950 million people to them (*ibid.*). Here the particularities of urbanisation on the continent have been a key focus of research, including the implications urbanisation has for particular diverse groups, such as women and youth (Meth et al., 2019; Thieme, 2018). Yet as recent scholarship has pointed out, while there has been a substantial growth in scholarship on the urban South and especially urban Africa (driven in large part by wider shifts including the rise of southern knowledges and epistemologies), there has yet to be much of a connection between research on urbanisation processes and its diverse implications and relationships to sexuality (Tucker, 2020; Tucker and Hassan, 2020).

Second, therefore, and closely following on from the first reason, is the historic connection studies of sexuality have had to the study of urbanisation, especially in the global North. As some of the earliest work on the relationship between sexuality and urban space explored, cities such as San Francisco, New York and London have been important for the study of sexuality due to the way they acted as sites of community, social solidarity, and the building of socio-sexual networks for non-heteronormative groups which in turn could help enable economic and political representation (Binnie, 1995; Castells, 1983; Knopp, 1987). While this article and this special issue are most clearly not arguing that processes or theoretical insights that emerged from the global North with regard to cities and sexuality have direct and wholesale applicability in the urban South, especially as they can sometimes erroneously assume a teleological progression towards a one-size-fits-all model of sexuality-based political representation and identity politics (Tucker, 2009), it is still nonetheless important to consider how cities may be important for our understanding of sexuality on the continent. We can consider this in terms of the ways in which cities have more broadly been understood to encompass a range of processes and outcomes, which may relate not solely to heteronormative subjects, but also to non-heteronormative subjects (albeit in potentially differing ways). Here we can consider how cities remain sites of large-scale migration, how they can allow for and can enable new social (re)configurations, how they can allow communities to build and find positions of safety and solidarity, and how they may also indeed result in forms of economic and other forms of enterprise. In all these instances, as with the study of urbanisation in Africa more broadly, the city is not simply a placeholder or a backdrop for other processes to take place on. Rather, the city needs to be seen as a meshwork of overlapping systems, becoming as much an active agent in those complex processes, helping to give shape to, challenge and enable the way different non-heteronormative groups and communities are able to navigate the urban form, as much as the city is a reflector of those processes (Hassan and Tucker, 2021; Parnell and Pieterse, 2014).

Through such a positioning of the urban in relation to sexuality, we therefore find two closely interrelated motivations as to why a study of African urban sexualities may be germane. First, while the African city has increasingly become a focus for scholarship, the study of diverse non-heteronormative groups and communities explicitly in relation to it has yet to be considered in great depth. This is perhaps surprising considering the wealth of material that has focused on the relationship between the urban and sexuality elsewhere in the world. Yet second, the study of the African city, especially through the growth of work on southern urbanism, has cautioned us of the need to think about African urbanisation in ways that do not automatically take as their starting point theories or approaches that emerged from elsewhere. Equally, therefore, we should be cautious of studying the relationship between cities and sexuality in ways that do not take account of the specificities of African urbanisation, or the ways in which non-heteronormative sexualities are understood and articulated more broadly on the continent. In other words, while we may wish to see a key gap in the study of African urbanisation at present—namely a relative lack of focus specifically on the relationship between cities and non-heteronormative sexualities—we should not assume that this gap can be filled by simply applying theoretical approaches and schemas from elsewhere to the continent. This is not to say that there will not be any utility to considering how theories that do emerge from elsewhere may productively be reconfigured in African contexts (indeed as outlined below regarding some of the submissions to the special issue, this may indeed be the case). It is, however, to suggest that a greater appreciation of the topic of African urban sexualities also opens up to us radically new ways of considering the diverse relationships between the urban and non-heteronormative groups and communities. This is because, on the one hand, African urbanisation (and sustainable development in Africa more broadly) cannot be sufficiently and solely explained by urban development pathways that have emerged elsewhere and in earlier times. On the other hand, this is because scholars need to be cognisant of the particular ways non-heteronormative sexualities are articulated in very different contexts to the global North.

In this sense, we may wish to consider the study of African urban sexualities—and that which is focused on non-heteronormative sexualities in particular—as still somewhat of an open field. While as outlined above, there have been a wealth of studies both of non-heteronormative groups and communities and on urbanisation in Africa over recent decades, we are yet to consider what a concerted effort to connect these two fields may look like. What is clear, however, is that such connection(s) may not necessarily follow the path of scholarship elsewhere on the world. The contributions in this special issue, summarised below, give testament to that. In this sense, we may wish to see a more concerted focus on African urban sexualities as both adding to broader academic interests in southern knowledges and in southern urbanism, while simultaneously expanding the scope by which existing scholarship on cities and sexuality is currently articulated.

While the bulk of this special issue is concerned therefore with looking forward, at the possibilities in the future for the study of African urban sexualities, the framing of this special issue also invites a reflection on an earlier text I wrote, which in a different historical moment attempted to think through what the study of African urban sexualities may mean. This is clearly not to say that *Queer Visibilities* should be held up as *the* way of conducting research on African urban sexualities. Indeed, it is absolutely clear that *Queer Visibilities* was of a particular time and a particular place that may not be reflective of where the field of African urban sexualities can or should be going. Rather, it is to highlight one way in which the uniqueness of urban processes in Africa (in this instance related to legacies of colonialism and in particular apartheid in South Africa) could inform and relate to the study of sexualities. In this way, the book’s theoretical framing could be seen both to critique and to extend (then) existing approaches to the study of sexuality and the study of the relationship between sexuality and urban space that were occurring elsewhere and primarily in the global North. It did so by setting into relief such theories through empirical analysis of the material constraints imposed by racist state policies on communities themselves. Because of this, the book was calling as much for the emergence of context-specific theories as it was calling for greater empirical work on the study of specifically non-heteronormative sexuality in urban environments. While, as mentioned above, *Queer Visibilities* focus on Cape Town clearly does not mean that its theoretical approach or empirical findings may have wholesale applicability in other locations on the continent, a summary of it as presented here is to suggest just one way in which we may wish to continue rethinking how to study the relationship between sexuality and urban space, and the relationship between theoretical framings, the importance of new empirical research, and the possibilities of drawing on diverse ways of knowing.

## Looking Back at *Queer Visibilities*

Queer Visibilities was published in 2009 at a time when there existed relatively far less work on non-heteronormative sexualities on the African continent and even less work that considered the urban in relation to sexuality there. Comparatively at the time, the book was therefore positioned primarily in relation to research and theories that had been explored elsewhere, primarily in the global North, and especially in urban centres of privilege. Its main theoretical insight was therefore to think through how and why urban sexualities scholarship that was at the time still dominated by studies of ‘gay spaces’ in cities such as San Francisco, New York, London could be critically engaged with by exploring a city elsewhere, a city in the global South, and a city with a very different colonial racist, classed and gendered history. This is not to say that the book was only attempting to critique existing theories and approaches from elsewhere, but that at the time it was that body of work, rather than scholarship and theoretical insights drawn from Africa itself, that still overwhelmingly dominated debates about urban sexualities.

Because of this imbalance in scholarship, the book drew on at the time implicit and ever-present theoretical assumption or underpinning within urban scholarship on sexuality, namely that of ‘the closet’ and the rigidity of one particular type of ‘heterosexual/homosexual binary’. It argued that ‘the closet’ metaphor, which was rightly seen to be foundational to much sexuality-based scholarship at the time, and the defining structure of gay oppression during the twentieth century in the global North (Sedgwick, 1990), was by no means universal. It went on to argue that the grip of ‘the closet’ metaphor could inadvertently lead scholars towards searching for—and finding—only particular types of sexual subject; subjects tethered to particular Western-centric notions of identity/behaviour ‘congruity’, particular notions of a teleological ‘coming out’ towards identity ‘authenticity’, and particular ways these identities related to particular types of neatly delineated ‘gay spaces’. By positioning ‘the closet’ as but one way of operationalising a ‘heterosexual/homosexual binary’, the book made the case that there may well be radically different ways in which individuals with same-sex desire are able to become visible to each other and to wider heterosexual societies across a city, and that such forms of visibility would be dependent of particular historical and material factors and constraints. These factors included most obviously in the post-apartheid city the history of racial discrimination and spatial segregation, persistent gross economic inequalities tied to spatial dislocation, the continued threat of the HIV/AIDS pandemic (this was long before biomedical technologies such as PrEP), and contemporary racism.

Having decentred the primacy of ‘the closet’, the book then went on to make the case for the idea itself of ‘queer visibility’. The concept itself has since travelled widely and evolved and been deployed in various ways some of which take it far beyond its original intent. However, at the time, the concept was developed specifically to address how scholars might want to examine very different ways in which  non-heteronormative identities emerge and become knowable in relation to different wider heteronormative structures rooted in different race-based (and other) histories and urban spaces, and crucially also, how these different forms of identity visibility interacted sometimes contentiously and sometimes productively with each other. These forms of visibility where, so the book argued, inherently queer because they had tremendous power to trouble and problematise each other. They were also queer because they highlighted the strategic, pragmatic and indeed sometimes playful ways in which individuals from different historically racially and spatially segregated communities had been and still were able to engage with often-times oppressive forms of sexuality-based regulation in inventive ways. To search for what was ‘queerly visible’ was therefore simultaneously to decentre the primacy of ‘the closet’ metaphor and the risks it posed by directing scholarship to look for only certain types of sexual subject, to root an exploration of identity-based difference in relation to an understanding of particular historical legacies and material constraints, and to consider the power and possibilities of cross-community awareness and interaction. The book was therefore an attempt to re-evaluate existing theories and reposition some of the assumptions about them, but to do so by drawing on long-term ethnographic empirical work into the lives, struggles and strategic forms of agency deployed by different communities across a post-apartheid city.

By looking across Cape Town at typologies of queer visibility drawn from each of the three main historically racially segregated populations of the city, namely among black African, Coloured and White men, the book drew a picture of diverse forms of non-heteronormative expressions in the city at the time. While not claiming to be representative of all different forms of non-heteronormative sexuality expression in the city (it was, after all only focused on select groups of ‘men’)—and this category itself in the context of some of the historical work within the book has rightly been reinterrogated since—see work by Ramsden-Karelse, (2022), it did nonetheless attempt to suggest one particular (and by no means universal) course for how to go about future research on the relationship between sexuality and urban space that decentred the primacy of global North ways of framing the sexualised city.

Looking back at Queer Visibilities now, rather than in the moment it was written, several reflections emerge. First, while the book at the time was framed in part as a counter to then existing hegemonic ways of conceptualising the relationship between sexuality and space which were dominant in scholarship at the time, the book might also instead now be framed more forcibly as a way of doing things differently. In other words, while the introduction to the book introduced the concept of queer visibilities as a way of critically engaging, countering, and setting into relief existing scholarship which implicitly or explicitly relied on the Western-centric ‘closet’ metaphor to function, today the concept of queer visibilities might be seen also as a way in which an understanding of material specificities and localised histories away from global North metropolitan centres of privilege can allow for different types of theorising from the South. The book might therefore generously be framed as existing within a far wider set of discourses associated with the then emergent southern turn—and specifically southern urban knowledge production. Second, the book’s focus on ethnographic research among marginalised communities who had historically not been a focus of scholarly attention at the time, namely black African and coloured queer men from historically segregated and marginalised spaces in Cape Town, can also be seen to point towards the continued existence of uncharted territories in research on sexuality and the urban in Africa. While, at the time the book came out, an engagement with such communities was seen as a key and original intervention, today such an engagement in a city such as Cape Town could almost seem prosaic. Yet just as importantly, it remains the case that both in cities such as Cape Town and perhaps more importantly in a wide array of cities across the continent numerous blind spots remain. Such blind spots can be framed both in terms of the types of sexual subject that need to be considered, and the ways in which such subjects engage with, help shape, and are in part defined by radically different types of urban space. Lastly, the book can also be seen to have existed within (and possibly been constrained by) an adherence to social science methods and approaches. While the book therefore was seen to help further existing social science—and specifically, geographical—ways of producing knowledge about groups who had not been the focus of research before, it also meant that its approach short circuited other ways in which we may wish to engage with the relationship between sexuality and the urban. Here therefore there is an opportunity to consider other academic disciplines and other styles of writing towards understanding the relationship between sexuality and cities. Taken collectively, such reflections help form the basis as to why this special issue was put together.

## Charting a Course for African Urban Sexualities Research Today

As outlined above, we may wish to see a range of reasons why a more concerted focus on the study of African urban sexualities, and specifically non-heteronormative sexualities, may be germane. We may also wish to see how such future studies may chart radically new courses of research interest that speak both to existing work on sexualities and existing work on urbanisation on the continent. Equally, however, the openness of the field means that there exist a very wide range of ways in which scholars interested in the urban and interested in sexualities may approach the connection between these two areas of scholarship. For some, there may well be a desire to explore new languages and new theoretical approaches. For others, there may be a desire to think through how existing theoretical languages or concepts that may have their primary roots in the global North may also be adapted and productively applied elsewhere. For others still, there may be an urgent need to think through what the urban means for certain communities and groups who have not been a focus of urban research attention up until now. And lastly, for other scholars, there may be a desire to explore the relationship between the urban and sexualities in ways that move beyond traditional social science approaches. As outlined below, the contributions to this special issue each in their own way speak to such imperatives, often in interconnected ways.

In terms of theoretical approaches, various contributors for the interventions that follow have explored how existing theories and new languages may be applied to the study of African urban sexualities. Perhaps most directly, Eddie Ombagi’s intervention, drawing on his earlier work Ombagi, (2019) on queer life in Nairobi, explores how we may want to critically interrogate unitary ideas of visibility and invisibility to instead consider how urban spaces can be inverted, subverted and reanimated in ways that allow for various queer subjectivities to emerge and connect with each other. Key to Ombagi’s intervention is how notions of queer ambivalence help us understand forms of queer liveability to help frame how subjects with same-sex desire can become known to each other while remaining unknown to others. For Ombagi, a framing therefore regarding a binary of visibility/invisibility negates an appreciation of the complex negotiations men with same-sex desire deploy as they navigate through and between different urban spaces in Nairobi, and the ways in which such negotiations are tempered by particular socio-spatial and temporal moments. Here we can also appreciate the need to consider the particularities of African cities. As Ombagi points out, Nairobi and Kenya, more broadly, have harsh social and legal prohibitions against homosexuality, which in part can be seen to have helped shape the forms of ambivalence deployed by men with same-sex desire there and the careful ways in which ‘archives of knowledge’ are created and shared between men. Such a reading and deployment of ambivalence as a socio-spatial tactic may well have utility in other cities on the continent; cities beyond the bounds of the South African state which remains an anomaly on the continent in terms of legal protections on the basis of sexuality but for which the majority of research still emanates.

Along a different trajectory, Gustav Visser presents a re-evaluation of the notion of homonormativity, a theoretical concept that has been deployed in various ways (primarily, of late in the global North) but which also emerged initially in South Africa through Visser’s earlier work. While the concept today is largely deployed to help articulate the exclusion of certain non-heteronormative subjects, often along raced, classed and gendered intersectional lines from certain spaces, and the overt visibility and privilege of white gay men, the term can also mean something slightly different. For Visser, homonormativity also needs to be understood in relation to the ways in which individuals with same-sex desire may be engaging in particular urban spaces in ways that break down neatly defined binaries, for example between ‘straight’ and ‘gay’. Drawing on historical reflections and recent developments in leisure space patronage during the COVID-19 pandemic in Stellenbosch, a secondary city 40 km away from Cape Town, Visser also reflects on the need for African urban research to move beyond an examination of large primary cities in relation to sexuality. This is necessary both to give voice to those inhabitants who live elsewhere and to consider the implications and impacts of spatial proximity to, but also potentially cultural dislocation from, primary cities where the majority of urban research more generally has been focused. Visser’s point here may well be key for future scholarship and especially future theorising on African urban sexualities; secondary cities currently and will continue to house the majority of urban residents on the continent (Andreasen et al., 2017).

Other contributors to this special issue, while still deeply enmeshed in various theoretical framings and debates, also bring to the fore the urgent need to consider diverse forms of sexual subject in relation to cities which have all but been ignored in scholarship until now. Zuziwe Khuzwayo explores the way black queer women navigate and engage with different types of urban space in Johannesburg. Pointing towards the continued imbalance in scholarship—both on the continent and further afield—which has tended not to focus on women with same-sex desire in relation to the urban, Khuzwayo explores a range of sites where black queer women have struggled to find places of acceptance and belonging. Khuzwayo also considers how these struggles feed into and perpetuate erroneous ideas that queer women and black queer women in particular in a city such as Johannesburg are simply ‘invisible’. Instead Khuzwayo’s intervention charts a variety of ways in which black queer women have made Johannesburg their home, considering how the city is at once presented as an urban utopia, a space that represents a constant threat of erasure, and a space that can enable innovate uses of the urban fabric to not only make do, but also to thrive. Here Khuzwayo’s exploration of *Pussy Parties* in Johannesburg as cultural events and forms of community building are set alongside other events emergent in other cities on the continent aimed at queer women, in places such as Cape Town, Durban and Nairobi, signalling the potential for a new set of research explorations into African queer urban geographies.

In a different, but still related manner, John Marnell’s intervention argues powerfully for more critical urban scholarship on the cross-border movements of LGBTQ Africans. As an example of an area of African sexuality-based scholarship that has seen significant growth over recent years but has not yet been set alongside and engaged with by urban scholarship, Marnell carefully excavates the multiple social and spatial dynamics at play in terms of the way queer and trans migrants navigate their (sometimes temporary) new homes. For Marnell there is an urgent imperative here; a focus only on macro level dynamics such as cross-border and transnational mobilities ignores the urban everyday in the lives of migrants themselves. By ignoring the everyday, potentially new and important avenues to theorise queer and trans displacement can be elided. In concert with other interventions in this special issue, Marnell also calls into question neatly defined binaries, which if implemented uncritically can fail to grapple with the complexity of, and agency inherent to, migrants’ lived experiences. Here Marnell’s intervention supports and also extends Ombagi’s intervention. While both explore Nairobi in terms of their ethnographic fieldwork, and both consider the need to appreciate the careful and strategic negotiations of LGBTQ groups to make the city their home which move beyond binaries such as visible/invisible, Marnell adds an additional dimension in terms of the fraught situations many migrants—and especially refugees—encounter in their day-to-day existence.

By shifting registers to consider the importance of the humanities, and in particular film studies, for research on sexuality and urbanisation, Xavier Livermon offers careful and nuanced consideration of the ways in which cities function more broadly in the spatial imaginaries of queer men in South Africa. Through a reading of the acclaimed film *Inxeba* about Xhosa initiation and the implicit (and explicit) relationship this rural ritual has to forms of male sexuality, Livermon is able to tease out how the urban—and specifically Johannesburg—remains deeply embedded in the spatial imaginaries and dreams of the film’s protagonists. As Livermon makes very clear, an articulation between male same-sex desire and the urban remains central to the film. Johannesburg, and the ways in which the city is seen by certain protagonists to enable forms of visible male sexuality at odds with a particular rendering of Xhosa tradition, becomes a placeholder for a wider set of processes seen to be eroding traditional culture. Yet at the same time, for some, Johannesburg is not simply a city to be feared, but also a city to be drawn to, despite the prohibitions some may experience due to their need to complete, and enable others to complete, Xhosa initiation rituals. While scholarship more broadly on urbanisation in Africa has highlighted large-scale migration to cities, Livermon reminds us that, even for those who do not migrate, the existence of the city remains central in how individuals make sense of their lives and create narratives about them.

The award-winning journalist and author, Mark Gevisser, then provides another unique way of exploring and articulating the link between the urban and sexuality in Africa. Drawing on his recent book *The Pink Line* (2020), which offers a global synthesis of sexuality-based struggles and experiences, and which was based on 7 years of empirical research, Gevisser’s engaging account explores how, specifically in urban Africa, spaces are being created for non-heteronormative individuals to express themselves despite often severe forms of social and legal sexuality-based discrimination. Gevisser also goes further and thinks through the connections between theoretical approaches from the global North to see what utility they may have—when reconfigured—in the urban South. Specifically, as Gevisser explains, the ‘Hirschfeld equation’ (drawing on the work of the sexologist Magnus Hirschfeld in Germany at the beginning of the twentieth century) may help us consider the power that the city holds both for anonymity and for socio-sexual connection. Here Gevisser can also be read alongside Ombagi and Marnell who collectively—but differentially—point out the power of the African city in relation to sexuality, due to its size and growth due to migration, its diversity of spatial forms, and the opportunities it therefore presents for new and inventive forms of sociality.

Lastly in this collection, Liza Cirolia and Andrea Polio invert the original framing of this special issue to consider how a queer disposition may support wider theorisation and empirical work on other areas of urban scholarship. While the majority of interventions in this special issue point towards a synthesis to more forcibly bring discussion of sexuality into discussion of the urban and urbanisation, for Cirolia and Polio there is utility in thinking how sexuality implicitly speaks to far wider urban debates. Drawing on Sara Ahmed’s work on queer phenomenology, Cirolia and Polio suggest the concept of ‘queer infrastructure’ as both an object of, and orientation towards, urban research practice. For Cirolia and Polio, therefore, sexuality is always ever present in urban research, even when not explicitly so. As both a theoretical intervention from the South and a call to action, Cirolia and Polio therefore highlight yet another way in which we may want to think through the current ‘open field’ of what future work on sexuality and the urban may mean for African cities.

## Conclusion

The interventions that follow hopefully stimulate and provoke debate. Collectively, they hopefully ask us to think about how theorising from the urban South can lead to new insights, or the retooling of existing theoretical apparatus. They also hopefully ask of us to appreciate the pressing need for further empirical scholarship on communities who have remained marginalised in the academy and especially within urban work. Lastly, they also gesture towards far broader ways in which we may wish to consider the relationship between sexuality and the urban that include but also extend beyond more ‘traditional’ social sciences approaches.

Yet it is also important, as with the summary of *Queer Visibilities* above, to appreciate that no one intervention or collection of interventions can sufficiently encompass the sheer diversity of experiences that exist at the interface between sexuality and the urban. The interventions that follow are but particular entry points aimed to stimulate future scholarship that is yet to occur. They are written by a particular subset of scholars, who hail primarily from South Africa, rather than the wider continent (even if they are writing about places elsewhere). As with *Queer Visibilities*, this special issue therefore is of a particular time—a time when South Africa remains dominant in work on sexuality on the continent, and when there remains an imbalance in who is conducting research about whom. Future scholarship, from more diverse places, and undertaken by more diverse voices, may well uncover other framings or equal or greater importance to those discussed here. (This is a topic returned to in the concluding summative article at the end of this special issue). What, however, may well remain important is that scholarship continues to think through emergent connections that bring into discussion sexuality and urbanisation on the continent.

## Footnotes

1 As noted in *Queer Visibilities*, see Brody and Potterat, (2003).
